# Outcomes of blended learning for capacity strengthening of health professionals in Guinea

**DOI:** 10.1186/s12909-021-02847-w

**Published:** 2021-07-28

**Authors:** Tamba Mina Millimouno, Alexandre Delamou, Karifa Kourouma, Jean Michel Kolié, Abdoul Habib Béavogui, Sara Roegiers, Marlon Garcia, Carlos Kiyan Tsunami, Stefaan Van Bastelaere, Wim Van Damme, Thérèse Delvaux

**Affiliations:** 1 Maferinyah National Training and Research Center in Rural Health, Forecariah, Guinea; 2 Africa Center of Excellence (CEA-PCMT), Faculty of Health Sciences and Techniques, Gamal Abdel Nasser University, Conakry, Guinea; 3 Department of Public Health, Institute of Tropical Medicine, Antwerp, Belgium; 4 Department of Clinical Sciences, Institute of Tropical Medicine, Antwerp, Belgium; 5 Belgian Development Agency (Enabel), Brussels, Belgium

**Keywords:** E-learning, Distance learning, Online learning, Web-based courses, Blended learning, Capacity strengthening, Health professionals, Guinea

## Abstract

**Background:**

Quality human resources constitute an essential pillar of an effective health system**.** This paper analyzes the outcomes of blended learning for post-Ebola capacity strengthening of health professionals in Guinea.

**Methods:**

Two courses lasting 3 months each (7–8 modules) were developed and implemented: one in Primary Health Care (eSSP) and the other in Sexual and Reproductive Health Services Management (eSSR). Both eSSP and eSSR courses were offered online on the Moodle platform, followed by a face-to-face capacity-building workshop. A cross-sectional study using a mixed-methods approach was conducted in 2018–19. As outcomes, we described learners’ sociodemographic characteristics, course completion and success, and perceptions of the courses and support from the instructors, analyzed the factors associated with learners’ successful completion and reported on learners’ feedback on their blended learning experience. Quantitative data were analyzed using the STATA 15 software, and qualitative data were analyzed through content analysis.

**Results:**

Overall, 282 health professionals were enrolled for both eSSP and eSSR courses. The completion rate was 69.5% (196/282). The success rate for learners who completed the courses was 80% (156/196), and the overall success rate for enrollees was 55% (156/282). The dropout and abstention rates were 22 and 9%, respectively. On both eSSP and eSSR courses, the success rate of women enrolled was higher than or equal to men’s. The success rate of medical doctors enrolled (53% for eSSP and 67% for eSSR) was higher than for other health professionals, in particular nurses (9% for eSSP) and midwives (40% for eSSR). Course type was associated with success (AOR = 1.93; 95% CI = 1.15–3.24). Most learners strongly agreed that the courses are relevant for targeted health professionals (81 to 150/150), pdf course materials are well-structured and useful (105/150), the content of the modules is relevant, comprehensible, and clear (90/150), self-assessment quizzes are helpful (105/150), summative assessment assignments are relevant (90/150), the course administrators and IT manager were responsive to learners’ concerns (90/150), they will recommend the courses to colleagues and friends (120/150).

**Conclusion:**

Two blended courses for capacity strengthening of health professionals were successfully developed and implemented in Guinea.

## Background

Blended learning that combines online and face-to-face teaching is a promising tool in medical education [1–4]. The flexibility of blended learning enables the online delivery of content to be combined with the best features of classroom interaction and live instruction to personalize learning, encourage thoughtful reflection, and individualize instruction across a diverse group of learners [5, 6]. Although traditional skills are needed to align teaching strategies with teaching/learning objectives, e-learning, which refers to providing instructional resources, activities, assessments, and feedback online, is a valuable addition to the teaching toolbox [4]. However, completing and dropping out from online courses remains a significant concern [7–9]. For example, a report on online delivery within France revealed high dropout rates (70–90%) for all audiences and levels and all online training types [10]. However, a study conducted in the United States of America (USA) found a higher completion rate (82%) [11]. As for the success rate, it is often low (17–48%), as reported by several universities specialized in e-learning, particularly in Thailand, India, the United Kingdom (UK), Switzerland, and France [12, 13]. There is limited evidence on e-learning or blended learning course completion and success rates when it comes to Africa. A recent evaluation of a blended learning course for building workforce capacity for effective use of health information systems in Namibia and Tanzania reported a 73% completion rate of e-learning modules [14]. Another assessment of a blended learning course on tuberculosis for front-line healthcare professionals in Ethiopia showed a higher completion rate (90%) of the online component [15].

In Guinea, the development of quality human resources for health (HRH), particularly in public health, is a priority for health authorities and their partners to strengthen the national health system [16–19]. The Ebola virus disease epidemic in 2014–2016 led the Guinean government to invest in HRH, and the Ministry of Health (MoH) recruited 2950 health professionals, including medical doctors, nurses, midwives, and community health workers, to overcome the deficit of HRH in rural areas [20]. Medical doctors were deployed in health centers (medicalization), and health district management teams (HDMTs) were strengthened. However, this massive recruitment raised the issue of capacity building (orientation and upgrading) of new health personnel.

Internet coverage has considerably increased in recent years countrywide (from 0.4% in 2010 to 33% in 2018) [21], including in rural areas. Despite improving connectivity, e-learning is still underdeveloped. In response to this, a blended learning approach, technically and scientifically supported by the Institute of Tropical Medicine (ITM), Antwerp, Belgium, was locally developed and implemented in 2018 in Guinea as an effort to support the country’s post-Ebola recovery plan. The program was led by the Maferinyah National Training and Research Center in Rural Health (CNFRSR) in collaboration with the Gamal Abdel Nasser University of Conakry (UGANC). It started with two courses adapted to the local health system context, one in Primary Health Care (eSSP) and the other in Management of Sexual and Reproductive Health Services (eSSR).

The current paper aims to analyze the outcomes of the first phase of this blended learning experience to strengthen Guinea’s health professionals’ capacity. As outcomes of this blended learning, the paper 1) describes learners’ sociodemographic characteristics, course completion and success, and perceptions of the courses and support from the instructors, 2) analyzes the factors associated with learners’ successful completion, and 3) reports on learners’ feedback on their blended learning experience.

## Methods

### Setting

This blended learning experience was piloted in Guinea, a West African country with approximately 12 million inhabitants (in 2018), 64% of whom live in rural areas [22, 23]. The Guinean health system has three levels: local (38 health districts), intermediate (eight health regions), and central (MoH) [24]. In 2018, the health personnel working under the authority of the Ministry of Health was 15,889, including 2476 medical doctors, 217 pharmacists, 5249 paramedical staff (nurses, midwives, biologists, laboratory technicians), 4933 administrative and technical staff, and 3014 community health workers [25]. The national education system is organized into pre-university, university, and technical and vocational training levels. Medical training is provided by one governmental university (UGANC) and two private ones (the Kofi Annan University and the La Source University). The training of paramedical personnel, in particular nurses, midwives, and community health workers, is mainly provided by health schools (public and private) under the supervision of the *Ministère de l’Enseignement Technique, de la Formation Professionnelle, de l’Emploi et du Travail*. The three aforementioned universities also train paramedical personnel. The CNFRSR was created on November 16, 1988, and attached to the MoH with namely the following aims: 1) participating in the continuing education of human resources for health nationwide, 2) undertaking operational socio-health research to make recommendations for improving public health status, 3) providing care to the population of Maferinyah, and 4) delivering practical training for medical students in primary health care (PHC) (Présidence de la république de Guinée: Décret No95/233/PRG/SGG portant attributions et organisation du centre de formation et de recherche en santé rurale de Maferinyah, unpublished).

### Blended learning course design and delivery

As part of this blended learning experience, a team from CNFRSR developed two courses whose outlines were designed using the ‘backward design’ model [26–28]. Seven modules were developed for the eSSP course (introduction to PHC, local health system, PHC data management, monitoring of a health area, quality and integration of PHC, Community health approaches, and Health promotion). Eight modules were developed for the eSSR course (Introduction to Sexual and Reproductive Health (SRH), Local Health System, SRH data management, monitoring of a health area, quality and integration of care in SRH, Community approaches in SRH, SRH Promotion, and Gender and rights in SRH). A detailed description of the training design and implementation process has been published elsewhere [29]. A team from CNFRSR was first trained in e-learning through a blended learning program at ITM. An ITM team provided technical and scientific support throughout the development process of the training. The courses targeted health professionals, including medical doctors, nurses, midwives, community health workers, public health technicians, and last-year medical school students.

Learners were selected based on the following criteria: profession (health personnel), current occupation, motivation to take the course, exposure to computer and internet, residence, and nationality. Although the courses were specifically designed for the Guinean health workforce, they were also opened to foreigners who met the selection criteria to test the scope of the courses and get some feedback from French-speaking participants residing in other African countries. Thus, the courses were implemented by cohort of 20 to 25 learners, including at most five who were foreigners or resided outside Guinea. One administrator for each course was responsible for developing the contents of the modules and mentoring over the course duration. Each week-long module had an ‘expert’ facilitator who was in charge of responding to learners’ questions in the discussion forum. Learners were given a catch-up week after the first four modules and another catch-up week after the last module. The learning platform (Moodle) was locally managed by information technology (IT) specialist supported by ITM staff. A local course coordinator led the program. A face-to-face capacity-building workshop for learners from the second and third cohorts of both courses was organized.

**Fig. 1:**
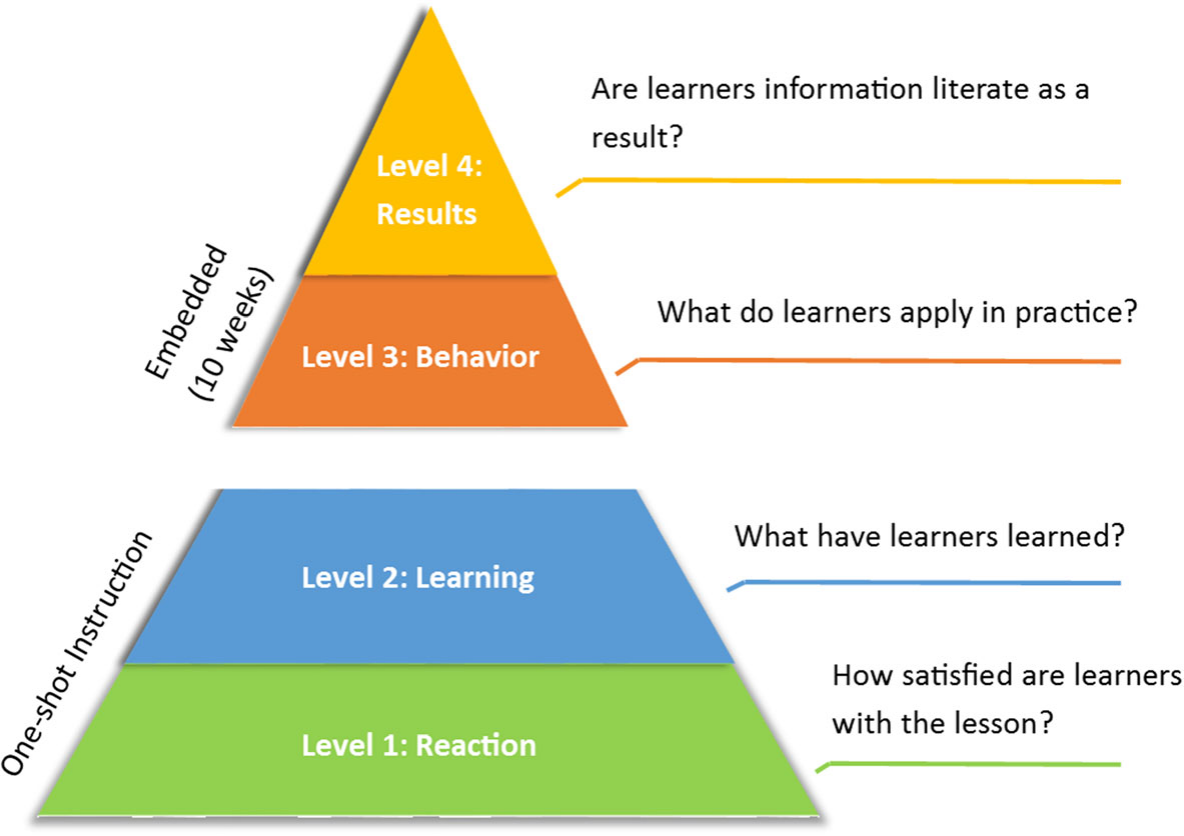
Kirkpatrick’s four-level training evaluation model [30]

The Kirkpatrick model (Fig. 1) was used for the training evaluation [30] and the results presented in this paper are based on levels 1 and 2 of this model.

### Study design and period

An evaluation of the implementation of the first phase (January 15, 2018, to January 15, 2019) of both courses (eSSP and eSSR) was conducted. It was a cross-sectional study using a mixed-methods approach.

### Study population and sampling

The quantitative strand included all enrollees (doctors, nurses, midwives, community health workers, public health technicians, and last-year medical school students) for both courses and all those who completed the courses and filled in the individual course evaluation form. Exhaustive sampling was therefore used for this quantitative component.

The qualitative strand focused on the learners who attended the face-to-face capacity-building workshop (after passing the online stage of the training). Only learners residing in Guinea were purposively selected to attend the workshop, and among these attendees, we interviewed those who consented.

### Data sources and collection procedures

The quantitative data was collected directly from learners’ selection databases and the results of the different cohorts. Additionally, the individual course evaluation form administered online at the end of each cohort was used for quantitative data collection. This course evaluation form included closed questions whose response options were framed following the Likert scale [31]. Finally, qualitative data was collected through learners’ interviews on the last day of the capacity-building workshop using an individual interview guide and authors’ personal reflection from the course design and implementation process.

### Quantitative data

Sociodemographic characteristics (age, sex, profession, residence, nationality, working time, course taken, and cohort) were collected. Course results (completion, success, dropout, and abstention) and learners’ perceptions of the courses and support from the instructors were compiled. Learners’ perceptions were collected of the following aspects: the relevance or adaptation of the courses for the targeted health professionals, the adaptation of the courses to the local health system, the structure and usefulness of pdf course materials, the relevance of the content of modules, comprehensibility of video presentations, helpfulness of discussion forum, helpfulness of additional learning materials, helpfulness of self-assessment quizzes, the relevance of summative assessment quizzes, the relevance of summative assessment assignments, the occurrence of technical problems for accessing learning materials, navigation on the online learning platform, course delivery accordance with the timetable, facilitators’ backgrounds and relevance of their responses to learners’ questions, the regular monitoring of learners by the administrators and their responsiveness to learners’ concerns, and the willingness of learners to recommend the courses to their colleagues and friends.

### Qualitative data

We collected learners’ feedback on their blended learning experience. The personal reflection focused on the lessons learned throughout the training and the challenges faced.

### Operational definitions

#### Completion rate

Is the number of learners who completed the course by performing all learning activities over the total number of enrollees.

#### Dropout rate

Is the number of learners who dropped out from the course after completing some activities over the total number of enrollees.

#### Abstention rate

Is the number of enrollees who ultimately did not log into the online learning platform although they had received all necessary information to get access over the total number of enrollees.

#### Success rate

Is the number of learners who passed the course (with an overall mean of marks greater than or equal to 5 out of 10) over the number of learners who completed the course (*success rate for learners who completed the course), and* over the total number of enrollees (*success rate for enrollees)*. Successful completion was used as the operational measure of success.

### Data analysis

#### Quantitative analysis

The data of the applicants and enrollees and the quantitative information retrieved from the course evaluation form were analyzed using the STATA software version 15 (Stata Corporation, College Station, TX, USA). Descriptive statistics were performed as proportions for categorical variables and as mean with standard deviation for continuous variables. We used Pearson’s chi-squared test and Fisher’s exact test to compare categorical variables and the Student’s t-test to compare continuous variables in the univariate analysis. A binary logistic regression was performed, and the odds ratios (OR; crude and adjusted) were calculated by considering the learners’ success as a dichotomous variable coded to 1 when the learner succeeded the course and 0 when he/she failed. All study variables with a *p*-value < 0.20 in the univariate analysis were included in the multivariate logistic regression. The significance level was set at 5%, with 95% confidence intervals.

#### Qualitative analysis

The qualitative data was analyzed using the content analysis approach.

The interviews conducted in French were recorded, then fully transcribed and translated into English manually. Then, we coded information into two categories (strengths and weaknesses) using inductive coding, which based on syntactic (keywords) and semantic (main ideas) analysis units. We processed data manually (semantic analysis) following interviewees’ main ideas and keywords and the resulting meaning [32, 33].

### Ethical considerations

The research protocol was approved by the National Ethics Committee for Health Research in Guinea (No: 022/CNERS/2020) and the ITM Institutional Review Board in Belgium (IRB Reference Code: 1363/20). Regarding the qualitative component of the study, free, informed, and oral consent was obtained from each selected participant before carrying out the interviews, and the data collected were coded. Both quantitative and qualitative data were only accessible to the research team. The database is stored on a computer protected by a password at the CNFRSR.

## Results

**Fig. 2:**
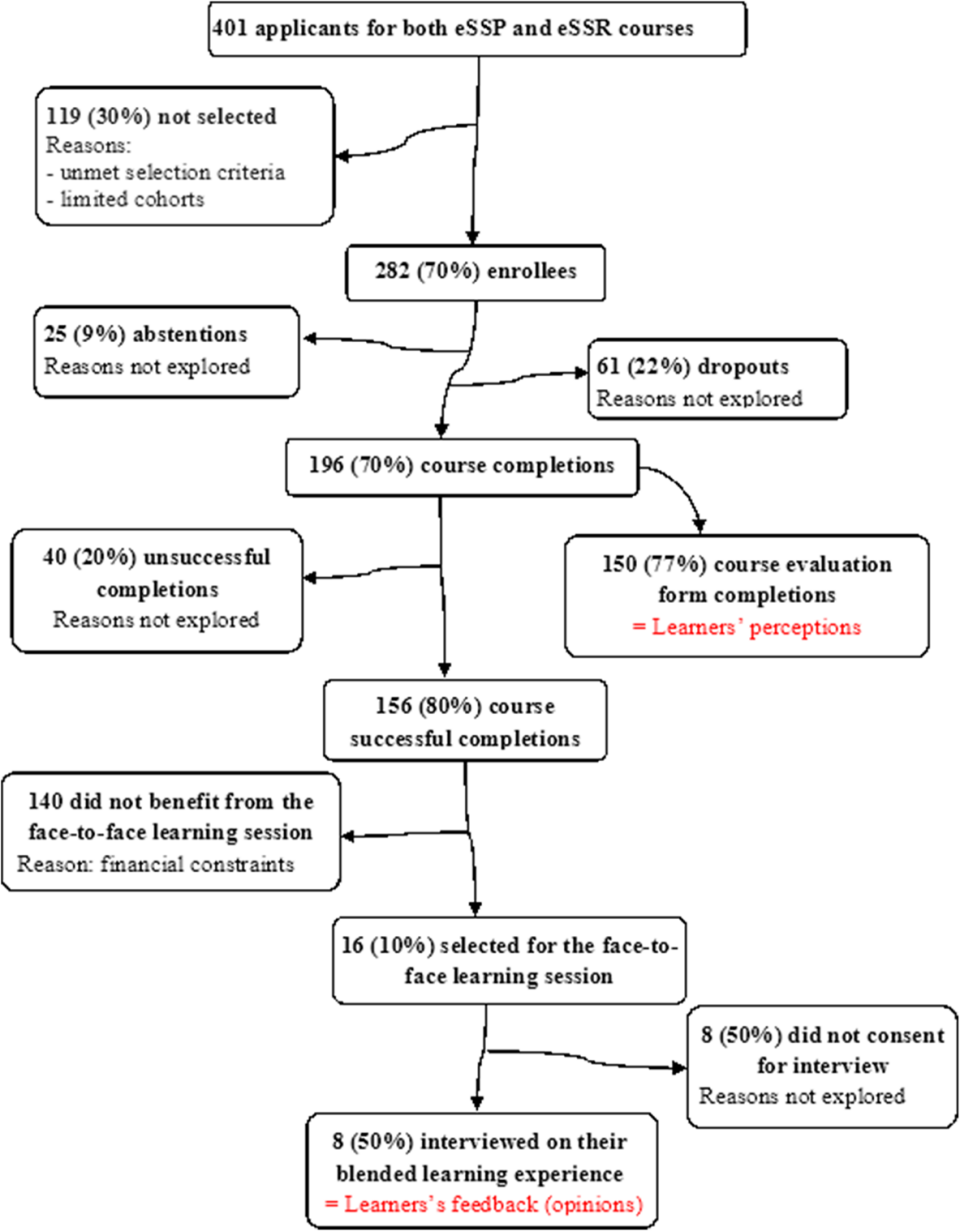
Participants’ inclusion flow chart on the blended courses in Primary Health Care (eSSP) and Management of Sexual and Reproductive Health Services (eSSR) and in the study, Guinea, 2018

Overall, 401 health professionals, including 94 (23.4%) women, applied for the courses (eSSP or eSSR) through a call for applications launched online at the end of December 2017 using the professional networks (District.Team Guinea’s mailing list [34], and medical, nursing and midwifery schools’ alumni networks), Facebook social network, and the instant messaging applications (WhatsApp and Messenger). A total of 282 learners were selected out of these 401 applicants (Fig. 2).

### Sociodemographic characteristics of learners

The 282 learners selected were enrolled for the training by the Maferinyah team. These 282 learners were collectively enrolled across six student cohorts per course.

Table 1 shows that learners’ ages ranged from 21 to 64 years for the eSSP course and 23 to 54 years for the eSSR course. For both eSSP and eSSR courses, the 30–40 years age group was the most represented (67.4%), and the mean age was 35 years (with standard deviations of 7.0 (eSSP) and 6.1 (eSSR)). Learners were primarily male (72.3%). As for the profession, medical doctors (77.6%) were the most numerous, followed by nurses (3.9%) and midwives (3.5%) (*p*<0.001). Foreigners by nationality were 20.6%, and enrollees who resided in countries other than Guinea at the time of the training were 20.2% (Table 1).

**Table 1:** Sociodemographic characteristics of learners on the blended courses in Primary Health Care (eSSP) and in Management of Sexual and Reproductive Health Services (eSSR), Guinea, 2018

Variables	eSSP course (***n*** = 135)		eSSR course (***n*** = 147)		Total (*n* = 282)		*p*-value
	Number	%	Number	%	Number	%	
**Age (years)**							
<30	22	16.3	24	16.3	46	16.3	1.000
30–40	91	67.4	99	67.4	190	67.4	
>40	22	16.3	24	16.3	46	16.3	
Mean (SD)	35 (±7.0)		35 (±6.1)				0.475
**Sex**							
Male	106	78.5	98	66.7	204	72.3	<0.001
Female	29	21.5	49	33.3	78	27.7	
**Profession**							
Medical Doctor	104	77.0	112	76.2	216	76.6	<0.001
Midwife	0	0.0	10	6.8	10	3.5	
Nurse	11	8.1	0	0.0	11	3.9	
Student (last year of medical studies)	9	6.7	12	8.2	21	7.5	
Other^a^	11	8.1	13	8.8	24	8.5	
**Nationality**							
Guinean	108	79.3	116	78.9	224	79.4	0.312
Foreign	27	20.7	31	21.1	58	20.6	
**Residence**							
Guinea (capital city, Conakry)	53	39.2	71	48.3	124	44.0	0.568
Guinea (other regions)	51	37.8	50	34.0	101	35.8	
Foreign country	31	23.0	26	17.7	57	20.2	
**Working time**							
Full	52	38.5	40	27.2	92	32.6	0.043
Part	83	61.5	107	72.8	190	67.4	
**Cohort**							
One	20	14.8	20	13.6	40	14.2	0.97
Two	23	17.0	25	17.0	48	17.0	
Three	25	18.5	24	16.3	49	17.4	
Four	24	17.8	26	17.7	50	17.7	
Five	24	17.8	26	17.7	50	17.7	
Six	19	14.1	26	17.7	45	16.0	

*SD* Standard Deviation

^a^Community health workers, Pharmacists, Biologists, Public health technicians

### Completion and success rates of/to the courses

Table 2 displays that of the 282 enrollees, 69.5% (196/282) completed the courses, and of these, 79.6% (156/196) passed the assessment assignments and obtained a successful completion certificate. The success rate for enrollees was 55.3% (156/282). For the eSSR course, the completion (70.7%) and success (89.4%) rates were significantly higher compared to those of the eSSP course, which were 68.2 and 68.5%, respectively (*p*<0.001). However, the overall dropout rate was 21.6%, and the overall abstention rate was 8.9% (Table 2).

**Table 2:** Results of learners on the blended courses in Primary Health Care (eSSP) and in Management of Sexual and Reproductive Health Services (eSSR), Guinea, 2018

Variables	eSSP course		eSSR course		Total		*p-*value
	Number	%	Number	%	Number	%	
	(*n* = 135)		(*n* = 147)		(*n* = 282)		
Completion rate	92	68.2	104	70.7	196	69.5	<0.001
Dropout rate	30	22.2	31	21.1	61	21.6	
Abstention rate	13	9.6	12	8.2	25	8.9	
	(*n* = 92)		(*n* = 104)		(*n* = 196)		
Success rate *for learners who completed the course*	63	68.5	93	89.4	156	79.6	<0.001
	(*n* = 135)		(*n* = 147)		(*n* = 282)		
Success rate *for enrollees*	63	46.7	93	63.3	156	55.3	<0.001

Table 3 depicts the results of learners by sex, profession, and residence. On the eSSP course, men had a higher course completion rate (74.5%) than women (65.5%); while on the eSSR course, men had a similar completion rate (70.4%) than women (71.4%). Physicians were the professional group with the highest completion and success rates, respectively 72.1 and 73.3% for the eSSP course and 71.4 and 93.8% for the eSSR course. Of the 11 nurses enrolled on the eSSP course and the 10 midwives enrolled on the eSSR course, only half of each professional category completed their course. Although women applied less for both courses and therefore represented a lower number and proportion of enrollments, their success rate on both eSSP and eSSR courses was either higher or equal to that of men (for the eSSP course, 51.7% of success rate for women vs. 45.3% for men and the eSSR course, the success rate was identical in both social categories (63.3%)). The physicians’ success rates for enrollees on both eSSP and eSSR courses (52.9% for eSSP and 67.0% for eSSR) were higher than that of other health professionals, especially nurses (9.1%) and midwives (40.0%). The eSSR learners were those who had the highest success rate (for enrollees) compared to that of the eSSP course (physicians: 67.0% for eSSR vs. 52.9% for eSSP; 40.0% among midwives for eSSR vs. 9.1% among nurses for eSSP; students: 58.3% for eSSR vs. 33.3% for eSSP). Most enrollees were from the capital city Conakry than other regions on both eSSP and eSSR courses. However, those from other regions had the highest completion rates (Table 3).

**Table 3:** Results of learners on the blended courses in Primary Health Care (eSSP) and in Management of Sexual and Reproductive Health Services (eSSR) by sex, profession, and residence, Guinea, 2018

Variables	eSSP course					eSSR course				
	Enrollees^1^ (*n* = 135)	Completion		Successful completion		Enrollees^1^ (*n* = 147)	Completion		Successful completion	
		Number^1^	%^1^	Number^2^	%^2^		Number^1^	%^1^	Number^2^	%^2^
**Sex**										
Male	106	79	74.5	48	60.8	98	69	70.4	62	89.9
Female	29	19	65.5	15	79.0	49	35	71.4	31	88.6
**Profession**										
Medical Doctor	104	75	72.1	55	73.3	112	80	71.4	75	93.8
Nurse/Midwife	11	5	45.5	1	20.0	10	5	50.0	4	80.0
Student	9	7	77.8	3	42.9	12	7	58.3	7	100.0
Other^3^	11	5	45.5	4	80.0	13	8	61.5	7	87.5
**Residence**										
Guinea (Capital city, Conakry)	53	38	71.7	28	73.7	71	47	66.2	43	91.5
Guinea (Other regions)	51	41	80.4	24	58.5	50	41	82.0	35	85.4
Foreign country	31	16	12.0	10	62.5	26	16	10.9	15	93.7

%^1^ = Number^1^/Enrollees^1^; %^2^ = Number^2^/Number^1^; ^3^Community health workers, Pharmacists, Biologists, Public health technicians

### Factors associated with learners’ success

The factors statistically significantly associated with learners’ success were sex (Crude OR = 1.22; 95% CI = 0.72-2.08; *p*<0.05) and the course taken (Crude OR = 1.96; 95% CI = 1.22-3.16; *p*<0.05) in univariate analysis. After adjusting the odds ratios in logistic regression, only the course taken remained statistically significantly associated with learners’ success (Adjusted OR = 1.93; 95% CI = 1.15-3.24; *p*<0.05). Indeed, learners who were enrolled on the eSSR course were about two times more likely to succeed than those on the eSSP course (Table 4).

**Table 4:** Factors associated with learners’ success on the blended courses in Primary Health Care (eSSP) and in Management of Sexual and Reproductive Health Services (eSSR), Guinea, 2018

Variables	Bivariate analysis			Multivariate analysis		
	Crude OR	95% CI	***p***-value	Adjusted OR	95% CI	***p***-value
**Age**						
<30	1 (ref)					
30–40	0.99	0.52–1.90	*0.980*	–	–	–
>40	0.77	0.34–1.75	*0.531*	–	–	–
**Sex**						
Male	1 (ref)					
Female	1.22	0.72–2.08	0.044*	1.11	0.59–2.10	0.734
**Profession**						
Other health professions^a^	1 (ref)					
Medical Doctor	1.78	0.76–4.17	*0.180*	1.47	0.60–3.60	0.393
Midwife	0.12	0.01–1.07	*0.058*	0.12	0.01–1.21	0.073
Nurse	0.78	0.17–3.52	*0.755*	–	–	–
Medical student	1.07	0.33–3.47	*0.905*	–	–	–
**Residence**						
Guinea (countryside)	1 (ref)					
Guinea (capital city)	0.95	0.56–1.62	*0.861*	–	–	–
Foreign country	0.59	0.31–1.14	*0.122*	0.76	0.36–1.60	0.480
**Working time**						
Part	1 (ref)					
Full	0.76	0.46–1.26	*0.295*	–	–	–
**Course**						
eSSP	1 (ref)					
eSSR	1.96	1.22–3.16	0.005*	1.93	1.15–3.24	0.014*
**Cohort**						
One	2.19	0.86–5.54	*0.098*	2.54	0.88–7.30	0.083
Two	1.33	0.57–3.07	*0.501*	–	–	–
Three	0.64	0.28–1.46	*0.294*	–	–	–
Four	0.79	0.35–1.78	*0.572*	–	–	–
Five	0.48	0.21–1.10	*0.085*	0.57	0.24–1.37	0.214
Six	1 (ref)					

^a^ = Community health workers, Pharmacists, Biologists, Public health technicians; *OR* Odds ratio; *CI* Confidence Interval; * = significant (< 0.05)

### Learners’ perceptions of the courses and support from the instructors during the course online component

Of the 196 learners who completed the courses, 150 (76.5%) expressed their perceptions of the courses and support from the instructors through the evaluation form as presented in Table 5.

**Table 5:** Learners’ perceptions of the blended courses in Primary Health Care (eSSP) and in Management of Sexual and Reproductive Health Services (eSSR), Guinea, 2018

Items (***n*** = 150)	Strongly Disagree	Disagree	Neutral	Agree	Strongly Agree
	n (%)	n (%)	n (%)	n (%)	n (%)
The courses are relevant for targeted health professionals:					
Medical doctors	0 (0)	0 (0)	0 (0)	0 (0)	150 (100)
Nurses and midwives	0 (0)	0 (0)	0 (0)	30 (20)	120 (80)
New health officials	0 (0)	0 (0)	0 (0)	0 (0)	150 (100)
Health facility and program managers	0 (0)	0 (0)	0 (0)	7 (5)	143 (95)
Other heath professionals^a^	12 (8)	33 (22)	14 (9)	10 (7)	81 (54)
The courses are adapted to the local health system	0 (0)	0 (0)	0 (0)	0 (0)	150 (100)
Pdf course materials are well structured and useful	0 (0)	0 (0)	0 (0)	45 (30)	105 (70)
The content of the modules is relevant, comprehensible and clear	0 (0)	15 (10)	0 (0)	45 (30)	90 (60)
Video presentations are understandable	30 (20)	60 (40)	0 (0)	15 (10)	45 (30)
The discussion forum was helpful	0 (0)	45 (30)	15 (10)	15 (10)	75 (50)
Additional learning materials are helpful	10 (7)	19 (13)	29 (19)	62 (41)	30 (20)
Self-assessment quizzes are helpful	0 (0)	21 (14)	18 (12)	6 (4)	105 (70)
Summative assessment quizzes are relevant	6 (4)	17 (11)	4 (3)	55 (37)	68 (45)
Summative assessment assignments are relevant	0 (0)	4 (3)	6 (4)	50 (33)	90 (60)
Learners encountered technical issues to access to learning materials (videos, pdf materials, quizzes, discussion forum)	75 (50)	15 (10)	15 (10)	45 (30)	0 (0)
Navigation on the Moodle platform is easy	0 (0)	45 (30)	0 (0)	30 (20)	75 (50)
The training agenda was followed as planned	0 (0)	0 (0)	0 (0)	0 (0)	150 (100)
Facilitators have strong background	0 (0)	45 (30)	15 (10)	15 (10)	75 (50)
Facilitators’ responses to learners’ questions were relevant	0 (0)	15 (10)	30 (20)	60 (40)	45 (30)
The administrators and IT manager were responsive to learners’ concerns	0 (0)	45 (30)	0 (0)	15 (10)	90 (60)
The administrators and IT manager regularly monitored learners’ progress	0 (0)	45 (30)	0 (0)	30 (20)	75 (50)
Learners will recommend the courses to colleagues and friends	0 (0)	0 (0)	0 (0)	30 (20)	120 (80)

*IT* Information Technology

^a^ = Community health workers, Biologists, pharmacists, public health technicians

### Learners’ feedback on their blended learning experience

Overall, 16 (10%) of the 156 learners who successfully completed the courses participated in the face-to-face capacity-building workshop. Among these 16 workshop attendees selected from the first and second cohorts, half agreed to be interviewed about their experience of both online and face-to-face learning sessions.

A participant from the eSSP course said: “*I found this training very interesting and met my expectations. This course was made up of rich and well-synthesized modules that actually motivated me to find out the next modules. I was amazed to see a platform where I navigated with great ease; I had the opportunity to see how I was evolving with this online training. I had access to my marks… It was an exciting training.*” (Cohort 2, male).

Another participant from the eSSR course stated: “*I found this training essential, truly relevant, and very qualified; it meets the needs at the operational level where I work. I am satisfied in many ways, as today, I can apply everything I learned from this course. For example, I was not familiar with the health pyramid, but I now know what to do with the module on the local health system. When I take up monitoring which is one of the main activities of a health district, this course allowed us to understand what monitoring is and where our country wants to go to, that is to say, towards the improved monitoring.*” (Cohort 3, male).

Learners reported that the face-to-face capacity-building session was an innovation in continuing education in Guinea and allowed fruitful interactions between course administrators, module facilitators, and learners. A participant from the eSSP course asserted that: “*I found this workshop enriching and beneficial; it allowed me to consolidate the teachings that I have assimilated and to clarify many dark areas during the online training.”* (Cohort 2, female).

A participant from the eSSR course provided further details: “*I had many difficulties on the monitoring module concerning many figures, but this workshop allowed me to know where the values come from and the mode of calculation with simplicity. For example, the updating of the population.”* (Cohort 3, female).

The only weakness mentioned by the learners was linked to the low participation or absence of some facilitators in the discussion forum.

### Lessons learned

Developing the modules consumes time and requires sufficient human resources to develop the contents, implement the course according to the model used, plan prompt and constant mentoring, and avoid leading parallel cohorts for the same course. As for the recruitment of learners, we have learned that it is best to ensure that the selected candidates (enrollees) are always available to take the course before starting the training to minimize abstention or dropout rates. Face-to-face sessions are essential for building learners’ capacity and obtaining their opinions to improve the quality of the course.

### Challenges

This blended learning faced some challenges. They are mainly: the low number of women applying for courses, in particular nurses and midwives; difficulties related to technological access; some facilitators’ non-timely or irrelevant participation or absence in the discussion forum to support learning; and the non-inclusion of all learners who succeeded in the online phase summative assessment in the face-to-face capacity-building workshop due to financial constraints.

## Discussion

To our knowledge, this blended learning solution is the first of its kind to be developed and implemented in Guinea in the health sector. It strengthened learners’ capacity according to them, particularly in monitoring a health area and on the organization and functioning of the local health system. Also, thanks to this solution, an e-learning team (course administrators/facilitators and IT manager) was trained and set up at the Maferinyah center.

We found a similar course completion rate (70%) with that of a blended learning course for building workforce capacity for effective use of health information systems in Namibia and Tanzania (73%) [14]. In contrast, the course completion rate found on a blended learning course on tuberculosis for front-line health care professionals in Ethiopia (90%) was much higher than ours [15]. Nevertheless, our findings aligned with those of studies that highlight avenues to enhance the completion rate on online courses, though several other studies reported pessimistic results [10, 35, 36].

The success rate for enrollees found in our study (55%) was higher than the success rates reported by Asian (Thailand and India) and European (the UK and Switzerland) universities specialized in online learning (between 17 and 48%) [12, 13]. In our context, several arguments could explain our findings. In particular, the type of approach (the cohort model) used for implementing the courses—with a limited number of learners (20–25 per cohort)—hence facilitating the course administrators and the IT manager to regularly and individually monitor the learners. In addition, the relevance and possibly the quality of the contents of the modules and learning activities and the learners’ motivation could also be factors involved in higher success rates. In contrast, the success rate for enrollees in our study was lower than the 83% success rate reported by ITM in 2015 for the Francophone cohort of the online course on antiretroviral treatment (eSCART). However, it is important to note that on this course, all learners who completed the course had the option of resitting the summative assessments in case of unsuccessful attempts [37]. Indeed, according to a report on benchmarks for e-learning in Africa, the success rate can be higher than 90% if the e-learning efficiency conditions are applied (simple, attractive, and easily accessible delivery method with user-friendly navigation; a variety of communication tools for synchronous and asynchronous interaction between facilitators and learners, and between learners themselves; quality content, motivating pedagogical approach with clear objectives, numerous and varied learning resources, continuous technical and pedagogical support to learners and facilitators; and ethical aspects and continuous evaluation system incorporated) [12].

Though women were significantly the least represented, they showed impressive engagement during the training, resulting in a higher success rate than men. Around 8 out of 10 women succeeded in the summative assessments on the eSSP course compared to 6 out of 10 men, while as many than men, almost 9 out of 10 women/men succeeded in the summative assessments on the eSSR course).

In terms of profession, physicians were more represented on both eSSP and eSSR courses. They recorded the highest completion and success rates compared to nurses and midwives, of whom only half completed the courses with weak success rates. Therefore, there is a need for identifying and introducing strategies to stimulate nurses and midwives to apply massively for courses while ensuring closer mentoring to improve their completion and success rates.

The course type influenced the success rate of learners; learners who were enrolled on the eSSR course were around two times more likely to succeed than those on the eSSP course. A recent survey reporting on student experiences of participating in five collaborative blended learning courses in Africa and Asia revealed that the course type was significantly associated with a more positively rated perception of the usefulness of the online component [38]. These two findings are similar, as a positive perception of a course may be a determinant of successful completion. In our context, sociodemographic characteristics included in the study were not significantly associated with success rates. However, individual motivation and other factors that were not included in our analysis, such as technical issues, may have played a role; this could also be related to the mentoring and facilitation, which differed between the two courses. In this regard, active interaction among learners–learners/instructors (course administrator/facilitators/IT manager) in the online discussion forum should be reinforced as one out of five learners poorly rated the added value of the discussion forum to their learning outcomes. Several studies support the notion that interaction among learners–learners/instructors improves learning outcomes [39–43]. Studies have shown that instructors’ immediacy behaviors can positively influence learners’ feelings, cognitive learning, engagement, satisfaction, and self-efficacy beliefs [43–45]. Since learners’ active participation in the online discussion forum is not guaranteed, instructors should encourage learners to participate. They should also respond to learners’ questions promptly [40]. Also, instructors should regularly read and reply to comments and post new questions or discussion topics. They should adopt as well immediacy behaviors adapted to the online learning context, such as giving students individual feedback, engaging in discussions on the message board or having (video) calls with students, posting an (informal) introduction at the beginning of the course, or a new module, and to post new announcements or commentaries regularly [38].

Most learners on our courses (6 to 10 out of 10) were satisfied with the training. Indeed, it is known that adequate instructional methods, support, instructor feedback, course structure, and design can facilitate learner performance and satisfaction [42, 46–49]. This learners’ satisfaction might explain the low dropout rate (22%) on our courses [11].

### Limitations

We acknowledge that this study has several limitations. First of all, it did not assess the impact of the training on learners’ everyday professional activities as this blended learning outcome. Secondly, the variables entered into the regression analysis have a narrow scope (for instance, the non-inclusion of poor internet access and relevance of course materials to job responsibilities); consequently, they could not fully explain the reasons of learners’ successful course completion. Thirdly, qualitative interviews conducted had limited scope, and participants who did not complete the courses were not interviewed. So, the reasons behind abstention and dropout were not explored. Furthermore, interviews were only conducted among some learners from the second and third cohorts. Even if a qualitative approach does not require a representative sample, opinions may differ for the other cohorts. Finally, further study is needed to explain abstentions and dropouts from the courses and assess the impact of the training on successful learners’ professional activities and their work environment as a whole.

## Conclusion

This blended learning for the capacity strengthening of health professionals was successfully developed and implemented in Guinea. According to learners’ feedback, it enabled capacity building, particularly in monitoring a health area and data management. It also built local institutional capacity (Maferinyah e-learning team) in designing blended courses, developing modules, and independently managing the learning platform.

## Data Availability

All data generated or analyzed during this study are included within this published article.

## Abbreviations

CEA-PCMT: Africa Center of Excellence for Prevention and Control of Transmissible Diseases
CNFRSR: Maferinyah National Training and Research Center in Rural Health
eSSR: Sexual and Reproductive Health Services Management
eSSP: Primary Health Care
HRH: Human Ressources for Health
ITM: Institute of Tropical Medicine
